# Phytochemical Characterisation of *Sorbus* Species: Unveiling Flavonoid Profiles Related to Ploidy and Hybrid Origin

**DOI:** 10.3390/plants14010119

**Published:** 2025-01-03

**Authors:** Emina Korić, Violeta Milutinović, Alma Hajrudinović-Bogunić, Faruk Bogunić, Tatjana Kundaković-Vasović, Irma Gušić, Jelena Radović Selgrad, Kemal Durić, Haris Nikšić

**Affiliations:** 1 Faculty of Pharmacy, University of Sarajevo, Zmaja od Bosne 8, 71000 Sarajevo, Bosnia and Herzegovina; irma.gusic@ffsa.unsa.ba (I.G.); kemal.duric@ffsa.unsa.ba (K.D.); haris.niksic@ffsa.unsa.ba (H.N.); 2 Faculty of Pharmacy, University of Belgrade, Vojvode Stepe 450, 11000 Belgrade, Serbia; violeta.milutinovic@pharmacy.bg.ac.rs (V.M.); tatjana.kundakovic@pharmacy.bg.ac.rs (T.K.-V.); jelena.radovic@pharmacy.bg.ac.rs (J.R.S.); 3 Faculty of Forestry, University of Sarajevo, Zagrebačka 20, 71000 Sarajevo, Bosnia and Herzegovina; a.hajrudinovic@sfsa.unsa.ba (A.H.-B.); f.bogunic@sfsa.unsa.ba (F.B.)

**Keywords:** *Sorbus*, flavonoids, hybridisation, liquid chromatography, mass spectrometry, apigenin derivates, luteolin derivates, polyploidy

## Abstract

The genetic, morphological and taxonomic diversity of the genus *Sorbus* is due to homoploid and polyploid hybridisation, autopolyploidy and apomixis, which also influence the production and diversity of secondary metabolites, especially flavonoids. The aim of this study was to investigate the relationships and variations of flavonoids in terms of hybrid origin and ploidy level between the parental species and their hybrid derivatives. The sampling design included leaf material of the following *Sorbus* accessions from ten natural localities: parental taxa (di-, tri- and tetraploids of *S. aria*; diploid *S. torminalis* and *S. aucuparia*) and their di-, tri- and tetraploid hybrid derivatives from crosses of *S. aria* × *S. torminalis* (subg. *Tormaria*) as well as the tetraploid *S. austriaca* and *S. bosniaca*, which originate from crosses of *S. aria* × *S. aucuparia* (subg. *Soraria*). We analysed the flavonoid profiles from the leaf fractions by LC-MS. A total of 23 flavonoids were identified, including apigenin and luteolin derivatives, which distinguish the hybrid groups from each other. This profiling highlights the distinctiveness of the *Tormaria* and *Soraria* accessions and emphasises the potential of the subg. *Tormaria* for further research on bioactive compounds in biological studies.

## 1. Introduction

Natural plant extracts contain different chemical profiles and corresponding biological activities with great application potential. In this regard, there is a growing interest in the discovery and production of plant extracts used in the pharmaceutical, cosmetic and food industries due to their bioactive compounds [1]. Since the biosynthetic pathway secondary metabolite formation varies within the same species due to different factors, a major challenge is to define all possible variability factors that may influence the composition of secondary metabolites [2]. The genus *Sorbus* L. is recognised as a highly valued source of bioactive compounds for the development of pharmaceutical, cosmetic and nutraceutical products [3] but has not yet been sufficiently explored.

The genus *Sorbus* (whitebeams, rowans and service trees) comprises tree and shrub species characterised by enormous genetic and morphological diversity. This diversity is the result of polyploidisation, hybridisation and apomixis [4,5]. Recurrent hybridisation between diploid species of *S. aria* (L.) Crantz, *S. aucuparia* L., *S. chamaemespilus* (L.) Crantz and *S. torminalis* (L.) Crantz and backcrossing of hybrid derivatives with their parental species resulted in about 190–200 recognised species inhabiting Europe [6,7]. The genus is traditionally divided into five subgenera: (subg.) *Aria*, *Chamaemespilus*, *Cormus*, *Sorbus* and *Torminaria* [8]. However, phylogenetic analyses recognise at least two monophyletic groups (*Sorbus*, *Cormus; Aria*, *Torminaria* and *Chamaemespilus*) [9,10]. Due to the obvious polyphyletic nature of the genus and extensive hybridisation between the subgenera members, the *Sorbus* genus is divided into independent genera [7], but the traditional name is still in use.

Most species are polyploid, triploid and tetraploid and reproduce by facultative apomixis [4,5,8,11]. Most polyploid species are endemics with a narrow geographic distribution, whereas the parental species are much more widely distributed throughout Europe [12]. While many aspects of *Sorbus* taxonomy [6,8,11], genetics [4,13] and reproduction [5,14], including phytochemical diversity, have been thoroughly studied in different parts of Europe, they are still understudied in the Balkans.

The remarkable genetic, morphological and physiological variability within the genus *Sorbus* is also reflected in the production and diversity of secondary metabolites [15]. Polyploidy and hybridisation are considered triggers that cause increased productivity of secondary metabolites and their diversity [16]. In particular, hybridisation is a driving mechanism that facilitates the production of secondary metabolites and restores their combinations [17,18].

Recent studies have revealed a great diversity and variation in the phytochemical composition of the different *Sorbus* species [19,20,21]. The genus is rich in flavonoids including anthocyanins [21], phenolic acids [22,23], triterpenes/tannins [24] and various other phytochemicals known for their antioxidant properties [25,26], anti-inflammatory [27], antimicrobial [28], antihyperlipidemic [29], antidiabetic [30], neuroprotective [31], hepatoprotective [24] and cardioprotective effects [32].

Flavonoids, as a group of secondary metabolites, are useful indicators of hybridisation and, in some cases, polyploidisation [33,34], but recent studies do not support flavonoids as indicators of polyploidy. In previous phytochemical studies, well-known flavonols such as quercetin and kaempferol and their glycosides, along with their methyl derivatives and less common C-flavonoids, have been detected in various parts or organs of *Sorbus* taxa, including the species examined in this study: *S. aria*, *S. torminalis*, *S. aucuparia*, and *S. austriaca* [19,23,35,36,37,38]. Many studies in the literature have focused on the flavonoid content in *Sorbus* fruits or inflorescences [23,36,37,38,39], with fewer studies on the bark and even more limited research on the leaves [22,25]. It is interesting that leaf material is the least used in flavonoid research, even though leaves contain a much higher content of polyphenolic components compared to flowers and fruits [22,25]. Most studies investigated the phytochemical composition of crude methanolic extracts from fruits, inflorescences and leaves, while only two reported detailed phytochemical analysis of their fractions [21,39]. In most studies, the plant material used for the investigation of polyphenolic profiles also originated from cultivated *Sorbus* accessions from botanical gardens and parks [39,40]. Numerous studies dealing with the qualitative and quantitative analysis of *Sorbus* the polyphenolic compounds were based on LC-UV/VIS spectroscopy [39] and the Folin–Ciocalteu method [41]. In contrast, LC-MS [21,22], UPLC-QTOF-MS [19,42] and UPLC-ESI_MS [3] have been used in a few studies to determine the phytochemical profile of *Sorbus* species.

The aim of this study is to provide the first insights into the leaf flavonoid profiles of *Sorbus* species from the Balkans (Bosnia and Herzegovina) using liquid chromatography-mass spectrometry (LC-MS). We analysed the diploid *S. aria* (subg. *Aria* Pers.), *S. aucuparia* (subg. *Sorbus*) and *S. torminalis* (subg. *Torminaria*) as well as the members of the hybridogenous subg. *Soraria* Májovský and Bernátova (*S. aucuparia* × *S. aria*) and *Tormaria* Májovský and Bernátova (*S. torminalis* × *S. aria*). We included tetraploid S. austriaca (Beck.) Hedl. and *S. bosniaca* Hajrudinović, Frajman, Schönswetter and Bogunić from the subg. *Soraria*), as well as di-, tri- and tetraploids from the subg. *Tormaria*. In addition, we also included tri- and tetraploid samples of *S. aria*. In particular, our aim was to (1) identify flavonoid markers that distinguish *Sorbus* accessions; (2) investigate the influence of hybridisation on the variation in the qualitative composition of flavonoids; and (3) determine the relationships between the ploidy level and the qualitative and quantitative content of flavonoid compounds.

## 2. Results

### 2.1. Ploidy Level

The ploidy level of 17 individuals from 10 locations was determined (See Material and Methods). The absolute genome size revealed three ploidy levels. Diploid values (2C = 2x) were found in *S. aucuparia* (2C = 1.43 pg), *S. aria* (2C = 1.44 pg), *S. torminalis* (2C = 1.44 pg) and *S. torminalis* × *S. aria* (2C = 1.43 pg). Triploids (2C = 3x) of *S. aria* had 2C= 2.11 pg and *S. torminalis* × *S. aria* had 2C = 2.18 pg. Tetraploid cytotypes (2C = 4x) were observed in *S. aria* (2C = 2.80 pg), *S. austriaca* (2C = 2.79 pg), *S. bosniaca* (2C = 2.80 pg) and *S. torminalis* × *S. aria* (2C = 2.74 pg). (See Material and Methods).

### 2.2. Mass Spectrometric Identification of Flavonoid Compounds

**Figure 1:**
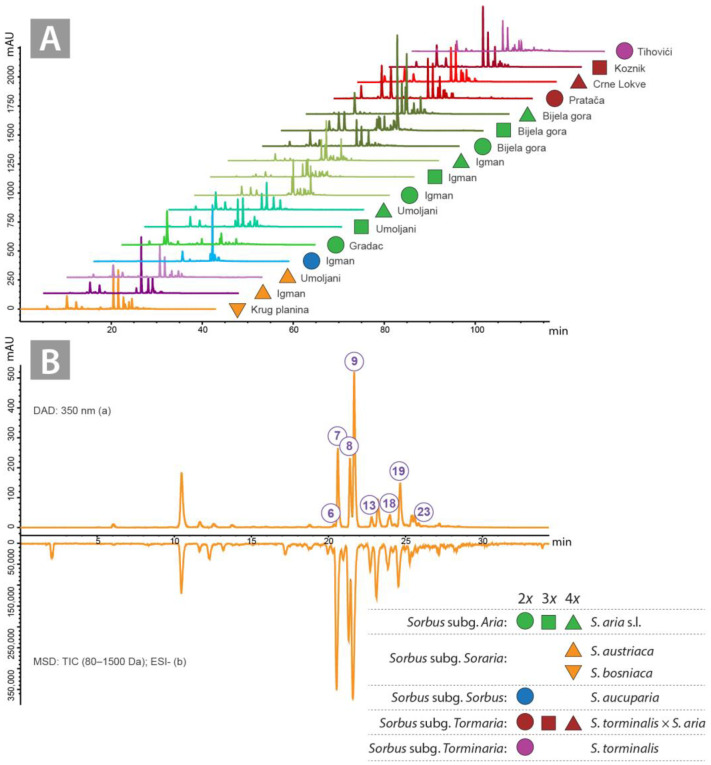
Liquid chromatography–mass spectrometry chromatograms (wavelength, 350 nm) of 17 analysed ethyl acetate fractions (**A**). The chromatograms are supplemented with localities for each analysed accession. and liquid chromatography–mass spectrometry chromatograms (a—diode array detector, b—mass selective detector) of the ethyl acetate fraction of *Sorbus bosniaca* leaves (**B**). Compound numbers are in circles and correspond to those in Table 1. The chromatograms are supplemented with localities for each analysed accession.

The structures of 23 flavonoids (**1**–**23**) were investigated using LC-MS by analysing 17 different ethyl acetate (EtOAc) fractions from methanol (MeOH) leaf extracts of various *Sorbus* accessions, including parental and hybrid individuals with different ploidy levels. The spectral data and the results of qualitative analyses are listed in Table 1, and the selected chromatograms are shown in Figure 1A,B. Ten compounds were identified after comparison with commercial standards: apigenin 6,8-di-*C*-glucoside (vicenin—**2**), apigenin-6-*C*-glucoside-8-*C*-arabinoside (schaftoside—**4**), quercetin 3-*O*-rutinoside (rutin—**7**), quercetin 3-*O*-galactoside (hyperoside—**8**), quercetin-3-*O*-glucoside (isoquercitrin—**9**), luteolin-7-*O*-glucoside (cynaroside—**10**), luteolin-7-*O*-glucuronide (**11**), kaempferol 3-*O*-glucoside (astragalin—**16**), quercetin 3-*O*-rhamnoide (quercitrin—**17**), apigenin 7-*O*-glucuronide (**21**). The structures of the other detected compounds were characterised based on similarities of the mass and UV spectra with level 3 data in the literature, according to Schymanski et al. [43].

#### 2.2.1. Flavones

Compounds **2**, **4** and **21** were assigned to apigenin glycosides based on their UV spectra (Table 1). The mass spectra (MS) of apigenin 7-*O*-glucuronide (**21**; [M–H]^−^ at *m*/*z* 445) contained signal corresponding to the aglycone at *m*/*z* 269 formed by the neutral loss of 176 Da, whereas flavonoids **2** and **4** showed different fragmentation patterns of *C*-flavonoid glycosides ([M–H]^−^ at *m*/*z* 593, and 563, respectively). As the *C*–*C* glycosidic bonds are more stable than *O*-glycosidic bonds, those flavonoids cannot yield fragments due to the loss of neutral sugar, but fragments resulting from the breakdown of the *C*–*C* bonds within the sugar moiety and total cross-ring cleavage [47,48]. In all three cases, the reference compounds confirmed the identity. The UV and MS spectra of **10**, **11** and **20** corresponded to luteolin glycosides (the MS spectra contained a fragment ion at *m*/*z* 285 corresponding to the aglycone). Compounds **10** and **20** showed a luteolin hexoside ([M–H]^−^ at *m*/*z* 447, fragment ion at 285), whereas **11** had a luteolin hexuronide structure ([M–H]^−^ at *m*/*z* 461, fragment ion at 285). The compounds identified as luteolin 7-*O*-glucoside (**10**) and luteolin 7-*O*-glucuronide (**11**) were confirmed with standard compounds.

#### 2.2.2. Flavonols

The UV spectra of flavonoids **1**, **3**, **5**–**9**, **12**–**15**, **16**, **17**, and **22** indicated the 3-substituted flavonol structure [49]. All mentioned flavonoids (except **12**, **16** and **22**) had MS spectra containing the signal of deprotonated quercetin molecule at *m*/*z* 301 (formed after sugar losses), and fragment ions from its RDA reactions [50]. Quercetin 3-*O*-rutinoside (**7**; [M–H]^−^ at *m*/*z* 609), quercetin 3-*O*-galactoside (**8**; [M–H]^−^ at *m*/*z* 463), quercetin 3-*O*-glucoside (**9**; [M–H]^−^ at *m*/*z* 463), and quercetin 3-*O*-rhamnoside (**17**) with the signal of the deprotonated molecule at *m*/*z* 447 were identified from standard compounds. Additional quercetin diglycosides **3**, **6** and **13** showed fragmentation patterns corresponding to quercetin dihexoside (**3**; [M–H]^−^ at *m*/*z* 625), quercetin deoxyhexosyl hexoside (**6**; [M–H]^−^ at m/z 609), and quercetin hexosylpentoside (**13**; [M–H]^−^ at *m*/z 595), respectively. The fragment ions were generated by the loss of one or more hexose units (162 Da), deoxyhexose (146 Da), or pentose moiety (132 Da).

The structure of compound **5** was assigned to quercetin trideoxyhexoside, while **14** was characterised as quercetin acetylhexoside (with signals of [M–H]^−^ at *m*/*z* 505, fragment ions at *m*/*z* 463 and 301), and **15** as quercetin pentoside ([M–H]^−^ at *m*/*z* 433, fragment ion at *m*/*z* 301). The kaempferol glycosides (**12**, **16**, **22**) were identified from the standard compound in the case of kaempferol 3-*O*-glucoside (**16;** [M–H]^−^ at *m*/*z* 447), or by analyzing their mass spectra. Therefore, **12** was characterised as kaempferol deoxyhexosylhexoside ([M–H]^−^ at *m*/*z* 593, fragment ion at *m*/*z* 285), and **22** ([M–H]^−^ at *m*/*z* 489) as acethylhexoside of kaempferol, based on the neutral loss of 42 Da corresponding to the acetyl group and the loss of hexose.

#### 2.2.3. Methylated Flavonols

Flavonoid glycosides **18**, **19** and **23** ([M–H]^−^ at *m*/*z* 477 and 519) showed mass spectra related to threehydroxy-methoxy-flavone-3-ol with fragment ions at *m*/*z* 316 (corresponding to aglycone) originating from neutral losses of hexose (in **18** and **19**) and acethylhexose (**23**); 301 (additional neutral loss of methyl group), 285 (additional loss of methoxy group).

### 2.3. Quantitative Analysis of Flavonoids

The quantification of the 10 identified flavonoids was performed using the external standard method with authentic commercial standards. Due to structural similarities, five of these standards were also used for the quantification of the remaining 13 identified flavonoids. The regression equations of the calibration curves, their correlation coefficients (r^2^), concentration ranges, LODs and LOQs are shown in Table S1. Secondary metabolites present in only one individual were not considered in the analysis. The amounts of flavonoids identified (in g/100 g DE) are listed in Table 2. Analysis of the ethyl acetate fractions revealed that quercetin flavonoids were the predominant component of the flavonoid profile in almost all studied *Sorbus* accessions (accounting for up to 75.34% of the total flavonoids detected in triploid *S. aria* from Mt. Igman). Luteolin flavonoids and apigenin flavonoids were detected only in samples of the subg. *Tormaria* group accounted for 6.5–17.8% and 1.7–60% of the total flavonoids identified, respectively.

Kaemferol flavonoids ranged between 1.3 and 21.8% and 2.0 and 3.5% of the total flavonoids detected in the subg. *Soraria* and *Tormaria*, respectively. Within the subg. *Soraria* group, a single triploid cytotype of *S. aria* from Umoljani is characterised by the trace/absence of kaempferol derivatives (0% for compounds **21** and **25** and a trace amount for compound **33**). Within the *Tormaria* group, a single triploid cytotype of *S. torminalis* × *S. aria* from Koznik was characterised by the complete absence of kaempferol derivatives.

### 2.4. Pattern of Flavonoid Variation and Relationships Among the Studied Sorbus Samples

**Figure 2:**
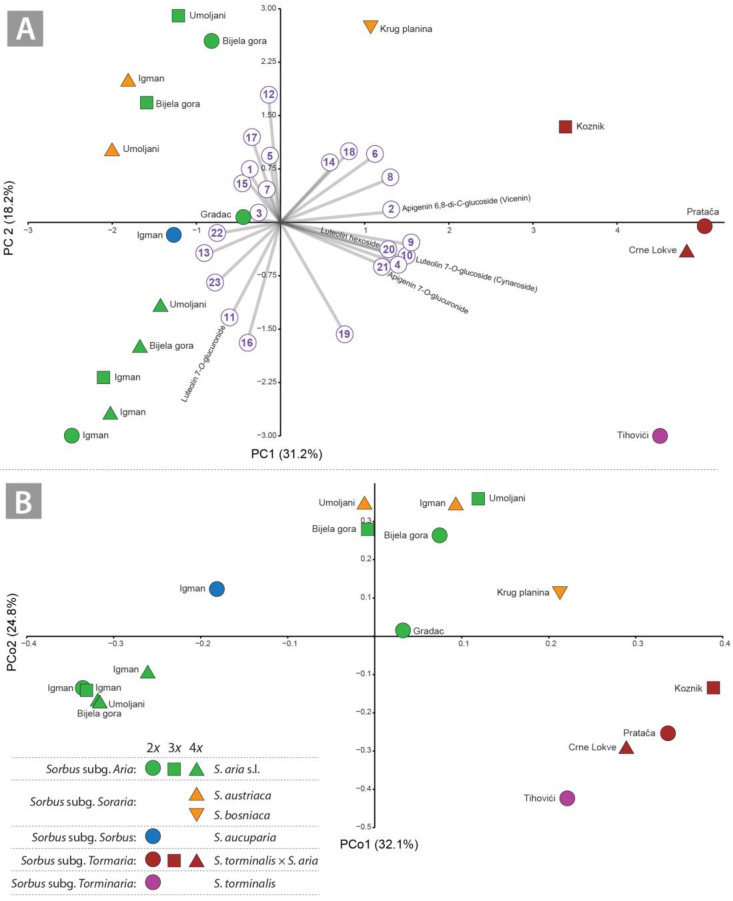
PCA and PCoA ordination ((**A**)—PC1 vs. PC2; (**B**)—PCo1 vs. PCo2) of the flavonoid compounds of the investigated *Sorbus* accessions. The ordination diagrams are supplemented with the localities for each studied accession. Compound numbers in circles (**A**) correspond to those in Table 1.

Phytochemical variation based on the quantitative data was complex and principal component analysis revealed eight significant principal components (Eigenvalues ≥ 1) (two components are shown). They accounted for 49.3% of the total variance (PC1 = 31.2%, PC2 = 18.1%; Figure 2A) and showed moderate correlation with most of the corresponding flavonoid compounds (Table S2). The following compounds contributed most strongly to PC1 (schaftoside, isoquercitrin, luteolin 7-*O*-glucoside and luteolin hexoside; luteolin 7-*O*-glucuronide, kaempferol deoxyhexosylhexoside, astragalin and methylquercetin hexoside isomer 2 contributed to PC2 (Table S2). The PCA ordination diagram showed the pattern in which *S. torminalis* and all *Tormaria* representatives along PC1 clearly diverged from the other *Sorbus* accessions (Figure 2A). The position of *S. bosniaca* was also divergent (Figure 2A). Along PC2, *S. austriaca* overlapped with a single diploid and two triploid *S. aria* samples separated from *S. aucuparia* and the remaining of *S. aria* samples, including all tetraploids (Figure 2A).

The PCoA analysis of all individuals showed a similar pattern as the PCA but with a higher resolution among the studied accessions (Figure 2B). *Sorbus torminalis* and all *Tormaria* members, as well as the diploids (Bijela gora and Gradac) and triploids (Bijela gora and Umoljani) of *S. aria*, *S. austriaca* and *S. bosniaca* are clearly distinct along PCo1 from *S. aucuparia* and all tetraploids of *S. aria* as well as the diploids and triploids of Mt. Igman. *Sorbus aucuparia* was separated from different *S. aria* cytotypes along PCo2 (Figure 2B). The members of the *Soraria* subg. (*S. austriaca* and *S. bosniaca*) and the cytotypes of *S. aria* were separated along PCo2 from the members of *S. torminalis* and *Tormaria* (Figure 2B).

**Figure 3:**
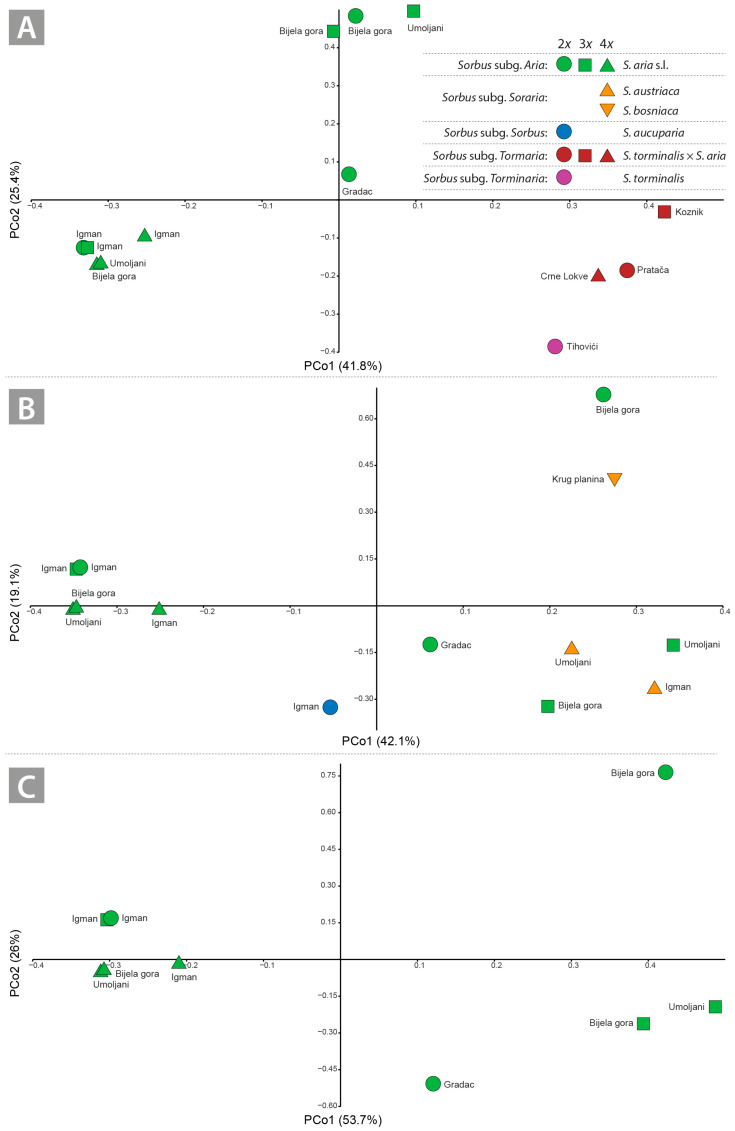
PCoA ordination (PCo1 *vs.* PCo2) of the flavonoid compounds of the investigated *Sorbus* accessions given for the subgenera *Tormaria* (**A**), *Soraria* (**B**), and *Aria* (**C**). The ordination diagrams are supplemented with the localities for each studied accession.

PCoA ordination of *Tormaria* members and putative parental species showed a clear position of *S. torminalis* and *Tormaria* cytotypes compared to *S. aria* cytotypes (Figure 3A). The cytotypes of *S. torminalis* × *S. aria* were clearly closer to *S. torminalis* than to *S. aria* (Figure 3A).

The ordination of *Soraria* samples (*S. austriaca* and S. *bosniaca*) showed an interesting pattern of spatial distribution between PCo1 and PCo2 (Figure 3B). Along PCo1, *S. bosniaca* and the diploid *S. aria* from Bijela gora diverged the most; the triploids of *S. aria* (Umoljani and Bijela gora) and the diploid *S. aria* from Bijela gora intermingled with *S. austriaca* compared to the tetraploids of *S. aucuparia* and *S. aria* and the diploids and triploids from Mt. Igman (Figure 3B). *Sorbus aucuparia* had an intermediate position along the PCo2 compared to the di-, tri- and tetraploid cytotypes of *S. aria* and *S. austriaca*. Along PCo2, *S. bosniaca* was clearly separated from the cytotypes of *S. austriaca* and *S. aria* (Figure 3B).

The most intriguing pattern was observed in the cytotypes of the *S. aria* (Figure 3C). The diploids of *S. aria* were the most divergent and separated along PCo1 and PCo2 (Figure 3C). While the tetraploid *S. aria* cytotypes represented a uniform group, the triploids segregated along PCo1. No specific pattern was recognisable for the *S. aria* cytotypes.

**Figure 4:**
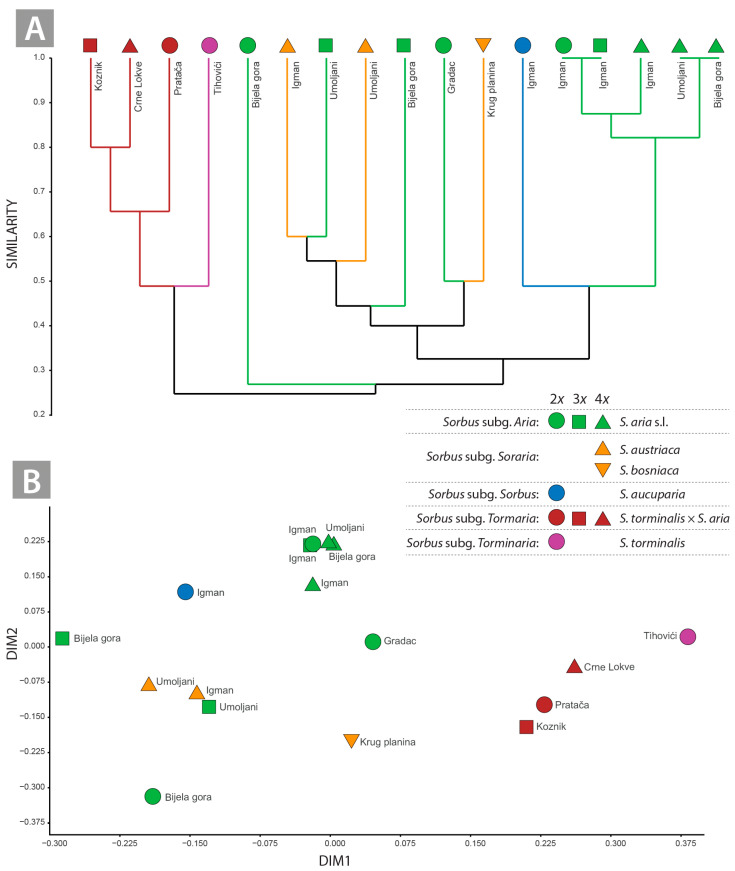
Results of multivariate analyses for 17 analysed *Sorbus* accessions. (**A**)—dendrogram showing the flavonoid relationships between the flavonoids of the accessions using the UPGMA method; (**B**)—non-metric dimensional scaling analysis of the studied accessions. Each figure is supplemented with localities for studied accessions.

The UPGMA cluster analysis revealed two main clusters (Figure 4A). The first cluster contained *S. torminalis* and its hybrids *S. torminalis* × *S. aria*. The second cluster contained two subclusters, one of which contained *S. bosniaca*, *S. austriaca*, *S. aria* diploids (Bijela gora and Gradac) and triploids (Umoljani and Bijela gora), while the second contained *S. aucuparia* and a group consisting of exclusively *S. aria* samples (Figure 4A).

The NMDS plot was consistent with the results of the previous multivariate analyses but showed a clearer separation between the *Sorbus* samples analysed (stress value S = 0.202 (Figure 4B). The relatively moderate stress value (S = 0.202) among the flavonoid compounds in the original data matrix is well illustrated in the ordination diagram (Figure 3B). The samples of *Sorbus torminalis*, *S. aucuparia*, *S. bosniaca*, the diploid *S. aria* from Bijela gora and the hybrid *S. torminalis* × *S. aria* differed significantly from each other. Di-, tri- and tetraploid *S. aria* cytotypes from Mt. Igman, Bijela gora and Umoljani were grouped together, while *S. austriaca* overlapped with the triploids *S. aria* (Figure 4B). Both the qualitative and quantitative values of the luteolin and apigenin derivatives clearly distinguished the *Tormaria* group, especially between the parental species *S. torminalis* and the closest cluster, which comprised hybrid derivatives of *S. torminalis* × *S. aria*. The active components that grouped di, tri and tetraploid individuals of *S. aria* from Mt. Igman, Bijela and Umoljani into a unique cluster were quercetin and kaempferol derivatives. In the differentiation of *S. aucuparia*, *S. bosniaca*, the diploid *S. aria* from Bijela gora, *S. austriaca* and the triploids of *S. aria*, predominantly quercetin derivatives were detected as discriminating metabolites.

The results of the Mantel test showed a weak and significant correlation between the Bray–Curtis distances (r = 0.285, *p* ≤ 0.004) for all studied accessions. In contrast, no correlation was found for the members of the subgenus *Aria* (r = 0.044, *p* ≤ 0.354).

## 3. Discussion

### 3.1. Novel Flavonoid Compounds in the Leaves of Sorbus Accessions

Our study reports the detection of 23 flavonoids in the EtOAc fractions from methanol (MeOH) leaf extracts of all *Sorbus* accessions examined, including 14 quercetin derivatives and three derivatives of apigenin, luteolin and kaempferol. This study is the first to analyse samples of triploid and tetraploid *Tormaria* accessions, *S. bosniaca*, and diploid and polyploid cytotypes of *S. aria* from the Balkan region. From the point of view of phytochemical research, this study demonstrated for the first time the presence of 14 flavonoids (Table 2) that had not been previously detected in the leaves of the *Sorbus* accessions studied. Among them, the rare flavonoids C-glycosides (apigenin 6,8-di-*C*-glucoside, apigenin-6-*C*-glucoside-8-*C*-arabinoside) were identified for the first time in representatives of the subg. *Tormaria*. Hydroxyquercetin deoxyhexosyl hexoside (**1**), quercetin trideoxyhexoside (**5**), quercetin deoxyhexosyl hexoside (**6**), quercetin hexosylpentoside (**13**), methylquercetin acetylhexoside (**23**), kaempferol deoxyhexosylhexoside (**12**) and kaempferol acetylhexoside (**20**) were found for the first time in *S. aria* and *S. austriaca*, while methylquercetin hexoside isomer 2 (**19**) was found exclusively in *S. aria* and quercetin pentoside in *S. austriaca*. For the first time, the presence of luteolin-7-*O*-glucoside (**10**), luteolin-7-*O*-glucuronide (**11**), luteolin hexoside (**20**) and methylquercetin hexoside isomer 2 (**19**) was detected in leaves of *S. torminalis*. In addition, this study is the first to show the flavonoid profile of leaves of different *S. torminalis* × *S. aria* cytotypes from the *Tormaria subg.* as well as the endemic species *S. bosniaca* from the *Soraria* subg. (Table 2).

Compared to the EtOAc fractions of *S. aria* leaves from the cultivars analysed in the study by Olszewska and Michael [51], the EtOAc fractions of *S. aria* leaves from the Bosnia and Herzegovina region contained higher amounts of the active components hyperoside (trace amounts in the study by Olszewska and Michael [51], up to 0.568 ± 0.006 g/100 g in our analysed dry extract) and rutin (1.37 ± 0.03 g/100 g in the samples reported by Olszewska and Michael [51], up to 7.504 ± 0.019 g/100 g in our analysed samples). On the other hand, the analysed EtOAc fractions contained lower amounts of astragalin and isoquercitrin compared to the EtOAc fractions of the cultivars from the study by Olszewska and Michael [51]. The EtOAc fractions of *S. torminalis* leaves from the natural habitat and the EtOAc fractions of *S. torminalis* leaves from Bosnia and Herzegovina analysed by Gunes AK et al. [21] are characterised by a high concentration of hyperoside (1.226 ± 0.014 g/100 g DE and 1.284 ± 0.009 g/100 g DE, respectively). Although quercitrin was not detected in the EtOAc fractions of *S. torminalis* analysed in our study, it was identified in the fractions analysed in the study by Gunes AK et al. [21]. In our study, methylquercetin hexoside isomer 2 (**19**) was detected for the first time in the EtOAc fraction of *S. torminalis* leaves at a concentration of 1.669 ± 0.015 g/100 g dry extract (DE).

### 3.2. Potential Discrimination of Subgenera and Lower Taxa in Sorbus: Influence of Flavonoid Profile on Hybridisation and Ploidy Level

Our analysis based on flavonoid profiles revealed intriguing relationships between the *Sorbus* accessions studied. Analyses based on quantitative data (PCA) and analyses based on binary data (PCoA, CA and NDMS) generally showed a similar pattern, although the latter provided a clearer resolution. The discrimination of the parental species *S. aucuparia* and *S. torminalis* is clear, in contrast to *S. aria*, which showed a remarkable divergence within the intragroup regardless of ploidy level and geographical origin (Figure 2A,B). The discrimination of subg. *Aria* and the hybrid subg. *Tormaria* and *Soraria* showed different patterns. While the members of subg. *Tormaria* are clearly diverge from each other and are more related to *S. torminalis* (Figure 2A,B and Figure 3A), the relationships in subg. *Aria* and *Soraria* are less clear (Figure 2A,B and Figure 3B,C). Indeed, the number of distinctive flavonoids detected in *Tormaria* was notably higher than in *Soraria* and *Aria* samples (Table 2). Moreover, certain compounds were only detected in *S. torminalis* and its hybrid cytotypes (Table 2), compared to the members of *Aria* (including all cytotypes) and *Soraria*. The large divergence between *S. torminalis* and *Tormaria* is primarily due to the presence of ‘discriminating’ compounds (apigenin 6,8-di-*C*-glucoside, luteolin 7-*O*-glucoside, luteolin 7-*O*-glucuronide, luteolin hexoside, apigenin 7-*O*-glucuronide).

The relationships between the parental species and the derived hybrids within the subg. *Tormaria* and *Soraria* are even clearer (Figure 3B,C). *Sorbus torminalis* acts as the maternal parent, as in most hybridogen taxa of the subg. *Tormaria* [4,13,52,53], suggesting a maternal effect in the inheritance of flavonoid compounds. The strong parental differentiation between *S. torminalis* on the one hand and *S. aria* on the other is due to the ‘discriminating’ compounds that are completely absent in the subgenus *Aria* (Table 2). These are derivatives of luteolin: luteolin-7-O-gluco-side (**10**), luteolin-7-O-glucuronide (**11**) and luteolin hexoside (**20**), characteristic compounds for the EtOAc fraction of *S. torminalis* × *S. aria* and the corresponding parental species *S. torminalis*. On the other hand, the derivatives of apigenin: apigenin-7-Oglucuronide (**21**) and apigenin-6-*C*-glucoside-8-*C*-arabinoside (**4**) are also characteristic compounds only for the EtOAc fraction of *S. torminalis* × *S. aria* and the corresponding parental species *S. torminalis*. The apigenin 6,8-di-*C*-glucoside (**2**) is exclusively present in the ethyl acetate fraction of *S. torminalis* × *S. aria*, but not in the parental species *S. torminalis*. In contrast to the previously mentioned observation, kaempferol 3-*O*-glucoside (**16**) is exclusively detected in the parental lineage *S. torminalis*, but is completely absent in the individuals of the hybrid *S. torminalis* × *S. aria.* The presence or absence of apigenin and luteolin derivatives allowed a clear distinction between the studied groups *Aria*, *Tormaria* and *Soraria*. These flavonoids, known for their biological activity, can serve as markers for the differentiation of the various *Sorbus* accessions. Within *Tormaria*, a complete absence of methylquercetin acethylhexoside (**23**) and quercetin pentoside (**15**) was found. Although the *Tormaria* accessions share most flavonoid compounds with the parental species, they also possess two exclusively novel compounds: (**21**) methylquercetin hexoside isomer 1 and (**1**) apigenin 6,8-di-*C*-glucoside (Table 2). Feulner et al. [53] found that the German *Tormaria* samples contained mixtures of the parental compounds; however, novel compounds were not detected.

The relationships between the *Soraria* samples and their parental species, particularly *S. austriaca* and *S. bosniaca*, were less clear (Figure 3B and Figure 4A,B). The samples of *S. austriaca* were more closely related to *S. aucuparia*, but surprisingly distinct from *S. bosniaca* (Figure 2A, Figure 3B and Figure 4A,B). The flavonoid composition of *S. austriaca* and *S. bosniaca* had only six common compounds (rutin, hyperoside, isoquercitrin, quercetin hexosylpentoside and kaempferol acetylhexoside, Table 2), whereas S. *bosniaca* also contains the three compounds found *in S. torminalis*, *Tormaria* subg. and *S. aria* cytotypes (quercetin deoxyhexosyl hexoside (**18**), methylquercetin hexoside isomer 1 (**21**) and methylquercetin hexoside isomer 2 (**22**), Table 2). In addition, the compound quercetin dihexoside (**3**) is found exclusively in *S. austriaca* and *S. aucuparia*, whereas it is completely absent in the subg. *Aria* and *S. bosniaca.* While the component quercetin acetylhexoside (**15**) is uniquely present in both samples of *S. austriaca*, quercetin trideoxyhexoside (**5**) and methylquercetin acethyhexoside (**23**) are detected in a single individual of *S. austriaca* (Mt. Umoljani). The absence of these compounds in the second *S. austriaca* sample is intriguing, as most Balkan populations represent a single genetic clone due to apomixis [54]. This finding suggests a possible independent origin of these two *S. austriaca* samples. In addition, a triploid *S. aria* sample (Mt. Umoljani) shares these two compounds with *S. austriaca* (Table 2), suggesting a possible hybridisation between the two species. The most common way to form *Sorbus* polyploids is by crossing a polyploid formation, whereas tetraploid apomict is crossed with a sexual diploid to produce a triploid apomict [5,55,56,57]. Therefore, such a scenario is likely, as the Umoljani site represents a large sympatric community of *S. aria* cytotypes and *S. austriaca,* which has been well studied in terms of ploidy dynamics and reproductive mode [57].

Similar patterns with respect to the presence or absence of quercetin derivatives are shown for methylquercetin acethyhexoside (**23**), quercetin deoxyhexosyl hexoside (**6**) and quercetin hexosylpentoside (**13**) in *S. austriaca* and *S. bosniaca* (Table 2). Such a variable composition of flavonoids in two closely related species that share at least one parental species may be due to several factors. First, both species originated from hybridisation between subg. *Aria* and *S. aucuparia* and represent the allotetraploid lineages, but their exact origin remains unclear [54,57]. In most taxa of the subg. *Soraria*, *S. aucuparia* is the maternal parent and the pollen donors are various members of the subg. *Aria*, whose identity remains unknown in most cases [4,14,52,55,57]. Therefore, the significantly different flavonoid profiles of these two species are not surprising (Table 2), which is due to the unknown paternal parent as well as the allopolyploid origin of these taxa. Each polyploid hybridisation is an independent event that can significantly restructure the genome, alter gene expression and induce physiological, phenotypic and biochemical changes [58,59]. Second, the diversity in flavonoid composition could be attributed to the parental influence of the subg. *Aria* members in hybridisation events, but this should be interpreted with caution and requires further research.

The effects of hybridisation on the occurrence of flavonoids differ within the subg. *Soraria* and *Tormaria*. The presence/absence of quercetin derivatives is associated with the subg. *Soraria*, whereas the variation of luteolin, apigenin and kaempferol derivatives is associated with the subg. *Tormaria*. In addition, *S. aria* as the paternal parent has a significant influence on the qualitative variation within the *Soraria* group in both subg., while *S. torminalis* plays a more important role in the subg. *Tormaria*. This phenomenon is probably also related to the biosynthetic pathway of the flavonoids. The pathway starts with p-coumaroyl-CoA and malonyl-CoA, which are condensed by chalcone synthase (CHS) to 2,4,6,4-tetrahydroxy chalcone. Calchone is then converted to naringenin by chalcone isomerase (CHI) [60]. From this point on, naringenin becomes a precursor for all the different biosynthetic pathways. The different biosynthetic pathways depend on the action of key enzymes such as flavone synthase (FSN) and flavonol synthase (FLS). When FSN is active, narigenin is converted into flavones. When FLS is active, narigenin is hydroxylated to form dihydroflavonols, which are precursors of flavanols [61]. Based on the above results and the absence of flavone derivatives in the *Soraria* samples, it can be concluded that the expression of the FSN enzyme is either repressed or inhibited, in contrast to the *Tormaria* samples. The question of whether the enzyme has been lost in the *Soraria* species or whether they have ever acquired it has not yet been clarified [62].

Certain individuals, both in the subg. *Soraria* and in the subg. *Tormaria*, produced novel compounds compared to the parental individuals. This is considered a rare example of hybridisation and polyploidy influencing the qualitative variation of secondary metabolites. Inheritance of secondary metabolites often conforms to Mendelian principles, where the presence of a secondary metabolite occurs when at least one parent produces it, resulting in complementary chemical patterns. However, deviations from this pattern are due to changes in the biosynthetic pathway [63]. These changes may include the addition or loss of genes or alleles that lead to new biochemical properties [17,63]. Blockages in the metabolic pathway can lead to the accumulation of intermediates, or unique interactions between enzymes and genes from each parent can lead to novel compounds [64]. Mutations in regulatory genes during hybridisation can also alter where or how secondary metabolites are produced, leading to greater biochemical diversity. On the other hand, the quantitative characteristics of secondary metabolites are controlled by multiple genes and influenced by dominant, recessive, additive or epistatic interactions [65]. F1 hybrids generally exhibit intermediate levels of secondary metabolite expression, with additive inheritance being the most common pattern. Additive effects account for 56% of the variation in secondary metabolite expression [17,65]. This variation can result from allelic differences affecting enzymes in the biosynthetic pathway or regulatory genes [17].

### 3.3. No Polyploid Effect on Flavonoid Profiles in S. aria Cytotypes

The production of secondary metabolites associated with ploidy is still a poorly understood aspect of plant biology. Polyploidy can lead to the differential regulation of biosynthetic pathways, affecting both the concentrations of biosynthetic products and the presence of certain metabolites. Studies indicate that tetraploids accumulate higher concentrations of phenylpropanoid compounds compared to diploid progenitors [66,67,68]. However, the concentrations of individual metabolites in polyploids can sometimes be lower than expected [68]. Our results, based on the Mantel test, generally showed a weak association of flavonoid profiles with the increase in ploidy in the *Sorbus* samples analysed. This pattern has been previously confirmed in other species [69,70,71,72,73]. On the other hand, no correlation was found between the increase in ploidy and the flavonoid profiles in samples of the subg. *Aria*. The *Aria* samples in this study exhibited a wide diversity of flavonoid profiles, regardless of ploidy level and geographical origin (Figure 3C and Figure 4A,B). The highest divergence among the diploid samples is particularly surprising. This pattern suggests that geographically determined flavonoid diversity is due to geographic origin [74]. Triploids and tetraploids showed less divergence (Figure 3C and Figure 4A,B), with the latter being the most homogeneous group within the analysed *Aria* group. The polyploids of the subg. *Aria* originated by hybridisation or polyploidy from diploid *S. aria* s. str. interacting with polyploids [8,75]. Therefore, the autopolyploid *versus* the allopolyploid origin of the *Aria* polyploids is probably also an important factor in shaping the flavonoid diversity within this group. The grouping pattern of *S. aria* tetraploids in this study was found to be the most homogeneous group in terms of flavonoid compounds (Figure 3C and Figure 4A; Table 2), suggesting an autopolyploid origin. It has been observed that the concentration of secondary metabolites increases in autopolyploids, probably due to a gene dosage effect [76,77], but this is not the case in the tetraploid samples.

In allopolyploids, it is assumed that the combination of biosynthetic complements from two progenitor species increases the variation in the resulting production of secondary metabolites [77]. Allopolyploidisation events can also lead to an organism that can produce all enzymes and metabolites of the progenitor species [78]. A high degree of divergence was observed in triploid *S. aria* samples (Figure 3C and Figure 4A,B), suggesting a possible allotriploid origin for at least two of them. This is particularly indicative of triploids of *S. aria* from Umoljani and Bijela gora, which intermix with *S. austriaca* (Figure 4A,B). Triploids of *S. aria* are most likely products of pollen exchange from apomictic tetraploids to sexual diploids, a pathway of triploid formation via the tetraploid bridge [5,9,57]. In the case of the two triploid *S. aria*, the potential pollen donor may be the tetraploid *S. austriaca* or *S. aria* [59]. The triploid *S. aria* from Mt. Igman is in the clade with the diploid and tetraploid *S. aria* samples from Mt. Igman (Figure 4A), indicating a probable autotriploid origin. The discovery of novel compounds in series of polyploid *S. aria* (methylquercetin acethylhexoside (**23**) in the diploid; hydroxyquercetin deoxyhexosyl hexoside (**1**) in the triploid; kaempferol 3-*O*-glucoside (**16**) and methylquercetin hexoside isomer 2 (**19**) in the tetraploid) suggests an allopolyploid origin of the triploids and tetraploids [69]. The comparison of the flavonoid profiles of *S. aria* from Mt. Igman shows that eight flavonoid compounds occur in all three ploidy levels. Of these, only one, quercetin-3-*O*-glucoside (**9**), shows an increase in the tetraploid sample, while other flavonoids do not follow the pattern of correlation between ploidy level and flavonoid content. In addition, a novel compound, quercetin 3-*O*-galactoside (**8**), was identified in the tetraploids. Of these, only one compound, quercetin 3-*O*-rutinoside (**7**), shows an increase in the tetraploid sample of *S. aria* from Bijela gora, while other flavonoids do not follow the pattern of correlation between ploidy level and flavonoid content. In addition, a novel compound, methylquercetin acethylhexoside (**23**), was found in the diploid *S. aria*, hydroxyquercetin deoxyhexosyl hexoside (**1**) in the triploid *S. aria* and kaempferol 3-*O*-glucoside (**16**), methylquercetin hexoside isomer 2 (**19**) in the tetraploid *S. aria* were identified.

## 4. Materials and Methods

### 4.1. Plant Material

Leaf material of *Sorbus* accessions (species and cytotypes) from 10 locations in Bosnia and Herzegovina was collected in July 2022 and prepared for phytochemical and cytometric analyses (Table 3). The identity of the studied taxa was confirmed by Faruk Bogunić and Alma Hajrudinović (Faculty of Forestry, University of Sarajevo, Bosnia and Herzegovina). The sampling design included the following *Sorbus* accessions: parental species (di-, tri- and tetraploids of *S. aria*; diploid *S. torminalis* and *S. aucuparia*) and their di-, tri- and tetraploid hybrid derivates from *S. torminalis* × *S. aria* crosses (*S.* subg. *Tormaria*) and tetraploid *S. austriaca* and *S. bosniaca* originated from *S. aria* × *S. aucuparia* crosses (*S.* subg. *Soraria*) (Table 3). The nomenclature system for the classification of subgenera followed Májovsky and Bernátová [79]. For this study, polyploid *S. aria* and *S. torminalis* × *S. aria* individuals were treated as cytotypes, as these entities have not yet been circumscribed. Detailed information of the geographic origin, genome size (GS) and ploidy level of the *Sorbus* accessions analysed is provided in Table 3. Fresh leaves were used for the cytometric analyses, while the remaining collected raw material was dried at a temperature of 20–25 °C and stored under dark, dry conditions.

### 4.2. Determination of Ploidy Level

Ploidy determination was preformed according to the protocol of Hajrudinović et al. [59] using flow cytometry. In brief, fresh leaves of selected individuals from 10 populations (Table 3) were co-chopped with fresh leaves of the internal standard, *Medicago truncatula* Gaertn. cv. R108-1 (0.98 pg, Marie and Brown [80], in 600 mL of cold Gif nuclear buffer [81]. The suspension was filtered through a 50 μm nylon sieve (CellTrics, Partec), and RNase (Roche) was added to 25 U mL^−1^. The cell nuclei were stained with propidium iodide (Sigma-Aldrich) at a final concentration of 50 mg mL^−1^ and incubated on ice for 5 to 10 min before analysis. The fluorescence of approximately 3000 cell nuclei was recorded for each sample using either a Partec CyFlow SL3 (Partec, Münster, Germany) 532 nm laser cytometer or a CyFlow Ploidy Analyser (Sysmex Europe SE) 532 nm laser. The 2C DNA values were determined and the DNA ploidy level [82] were derived by comparing these values with those of individuals with known chromosome number [83].

### 4.3. Sample Preparation for LC-MS Analysis

Dried leaves of *Sorbus* accessions were pulverized and extracted with methanol, which served as a suitable extraction solvent at 20 °C, with two cycles of 20 min each using ultrasound. The drug-to-solvent ratio was 1:40. The methanolic extracts were centrifuged at 4000 rpm for 20 min at 4 °C. The prepared methanolic extract was evaporated to dryness, reconstituted in water at a 1:100 ratio, and then subjected to successive liquid–liquid extractions with solvents of increasing polarity: (a) hexane, (b) diethyl ether and (c) ethyl acetate. The ethyl acetate fractions (EtOAc) were collected, evaporated to dryness, and dissolved in 1 mL of HPLC-grade methanol prior to analysis [84].

### 4.4. LC-MS Analysis

LC-MS analysis of EtOAc fractions of *Sorbus* leaves obtained from MeOH extracts was performed on an Agilent LC/MS system 1260/6130 (Agilent Technologies, Waldbronn, Germany) with diode array detector (DAD) and single quadrupole API-ESI MSD. A Zorbax SB-Aq column (150 × 3.0 mm; 3.5 μm particle size, Agilent Technologies), and a binary mobile phase (A—formic acid, 0.1%, *v*/*v*; B—acetonitrile) were used for chromatographic separation, and the gradient program, column temperature, flow rate, injection volume and DAD and MSD parameters were determined based on previous work [85]. The obtained dry fraction/extracts were dissolved in MeOH (final working concentrations were 5 mg/mL) and filtered through a cellulose membrane filter (0.45). The recorded raw data were processed using ChemStation (Rev. B.04.03-SP1) software. The external standard method was used for quantitative analysis and the levels of the detected compounds were calculated based on the peak areas obtained by DAD (at 350 nm) using calibration curves obtained for ten commercial standards (regression equations, correlation coefficients (*r*^2^), linear ranges, LODs and LOQs in μg/mL are given in the Table S1. Following the guidelines of the International Conference on Harmonization (ICH, 2005), the LODs and LOQs were determined using the signal-to-noise ratio as described in the European Pharmacopoeia (Ph. Eur. 11.0) [73,86]. The analysis was performed in triplicate. The amounts of the detected compounds were calculated as gram equivalents of the standards used per 100 g of dry fraction (dw) due to their high structural similarity.

### 4.5. Chemicals and Reagents

LC-MS-grade formic acid and acetonitrile, as well as standards (of HPLC purity): Rutin, Isoquercitrin, Quercitrin, Luteolin 7-*O*-glucoside, Luteolin 7-*O*-glucuronide, Astragalin, Apigenin 6,8-di-*C*-glucoside (Vicenin), Apigenin-6-*C*-glucoside-8-*C*-arabinoside (Schaftoside) and propidium iodide (Sigma-Aldrich, Merck Group, Merck KGaA, Darmstadt, Germany), Quercetin 3-*O*-galactoside (Carl Roth, Karlsruhe, Germany), Apigenin 7-*O*-glucuronide from HWI Analytik (Ruelzheim, Germany).

### 4.6. Data Analysis

Descriptive statistics (mean and standard deviation for each of the analysed compounds at the individual level), univariate statistics (one-way ANOVA followed by a Tukey HSD test) and multivariate techniques, including Principal Component Analysis (PCA), Principal Coordinate Analysis (PCoA), non-metric multidimensional scaling (nMDS) and Cluster Analysis (CA) were applied to assess the variation of flavonoid compounds. Triplicate measurements were averaged for each individual to create a data matrix. To reduce the magnitude of differences in the original values (the amounts of compounds expressed as relative percentages) and to avoid making assumptions about the data distribution, the data were coded (using categorical scale) following Milutinović et al. [87]. The aim of the PCA was to reveal a general pattern of variations and relationships between all individuals. To hypothetically reduce the influence of environmental factors on the production of flavonoid compounds in studied samples, we generated a binary data matrix for the presence/absence of compounds. We then performed separate PCoAs for all individuals, for the *Tormaria* and *Soraria* groups, including their putative parental species and their hybrid members (species and cytotypes), and for the *Aria* group. The PCoAs based on Jaccard distances were used to visualise the relationships between individuals. The cluster analysis by the unweighted pair-group method using arithmetic averages (UPGMAs) using Jaccard distances was performed to verify the affinities between samples. Finally, NMDS using the Bray–Curtis pairwise distance matrix was used to order the studied samples. The Mantel test [88] was used to check whether the quantitative composition of the flavonoid components is affected by the level of ploidy for all accessions, particularly for the *Aria* subgenus. The Bray–Curtis distances for raw phytochemical data and ploidy data were used for calculation. All analyses were performed using PAST 3.14 [89].

## 5. Conclusions

The results of HPLC-MS and flow cytometry proved valuable for the analysis of ploidy-dependent plant systems and the identification of flavonoid profiles in a panel of *Sorbus* accessions, including three diploid parental species and polyploid derivatives of the subg. *Tormaria*, Soraria and *Aria* from the region of Bosnia and Herzegovina. Of the 23 flavonoids identified, 14 were newly reported, improving the understanding of the phytochemical profiles of *Sorbus* species. We have shown that certain fractions of flavonoids are reliable indicators of hybridisation in certain *Sorbus* groups (*Tormaria* and *Soraria*). Correlation with ploidy levels reveals a non-statistically significant relationship with flavonoid profiles in a range of *S. aria* polyploids. Flavonoid profiling highlights the *Tormaria* group as target accessions rich in bioactive compounds for further biological studies.

## Figures and Tables

**Table 1:** UV and MS spectral data of the detected flavonoids in EtOAc fractions of *Sorbus* accessions.

No	Rt (min)	*λmax* (nm)	MW	*m*/*z* [M–H]^−^	Product Ions	Compound
Flavones						
**2** *	15.07–15.94	270, 338	594	593	226; 473	Apigenin 6,8-di-*C*-glucoside (Vicenin) ^st^ **
**4**	18.76–19.12	270, 338	564	563	473	Apigenin-6-*C*-glucoside-8-*C*-arabinoside (Schaftoside) ^st^
**21**	25.44–25.64	268, 338	446	445	269	Apigenin 7-*O*-glucuronide ^st^
**10**	22.22–22.48	254, 266sh, 350	448	447	285	Luteolin 7-*O*-glucoside (Cynaroside) ^st^
**11**	22.47–22.74	254, 266sh, 350	462	461	285	Luteolin 7-*O*-glucuronide ^st^
**20**	24.78–25.08	248, 268sh, 340	448	447	285	Luteolin hexoside [44]
Flavanols						
**1** *	14.99–15.12	252, 266sh, 372	626	625	317	Hydroxyquercetin deoxyhexosyl hexoside [45]
**3**	17.36–17.76	256, 264sh, 354	626	625	301	Quercetin dihexoside [23]
**5**	19.97–20.19	256, 264sh, 354	740	739	447; 593	Quercetin trideoxyhexoside
**6**	20.15–20.69	256, 264sh, 354	610	609	301	Quercetin deoxyhexosyl hexoside [45]
**7**	20.40–20.73	256, 264sh, 354	610	609	301	Quercetin 3-*O*-rutinoside (Rutin) ^st^
**8**	21.28–21.57	256, 264sh, 354	464	463	269; 301	Quercetin 3-*O*-galactoside (Hyperoside) ^st^
**9**	21.50–21.96	256, 264sh, 354	464	463	269; 301	Quercetin-3-*O*-glucoside (Isoquercitrin) ^st^
**12**	22.67–23.10	266, 348	594	593	285	Kaempferol deoxyhexosylhexoside [45]
**13**	22.61–22.96	256, 264sh, 354	596	595	301; 433	Quercetin hexosylpentoside [46]
**14**	22.51–23.38	256, 268sh, 356	506	505	301; 463	Quercetin acetylhexoside [22,46]
**15**	23.88–24.08	256, 264sh, 354	434	433	301	Quercetin pentoside [22]
**16**	24.00–24.23	266, 348	448	447	285	Kaempferol 3-*O*-glucoside (Astragalin) ^st^
**17**	23.98–24.30	256, 264sh, 354	448	447	301	Quercetin 3-*O*-rhamnoide (Quercitrin) ^st^
**22**	25.80–26.27	266, 348	490	489	285; 447	Kaempferol acetylhexoside [46]
Methylflavonols						
**18**	23.85–24.17	256, 268sh, 356	478	477	271; 301; 315	Methylquercetin hexoside isomer 1 [46]
**19**	24.26–24.80	256, 268sh, 356	478	477	271; 285; 301; 315	Methylquercetin hexoside isomer 2 [46]
**23**	25.98–26.30	256, 268sh, 356	520	519	285; 301; 316; 447	Methylquercetin acethylhexoside [46]

* The numbers of the detected compounds, based on the order of elution; Rt—retention time; ** The references for comparison of the UV and/or MS data of the compounds are given in square brackets; st.—commercial authentic compounds used for identification; sh.—second harmonic wavelength.

**Table 2:** Quantities (%, g/100 g DE) of detected flavonoids in EtOAc fractions of *Sorbus* accessions.

Taxon	Subgenus	Location	DNA Ploidy Level	Flavones													
				Xsr (%)		STD		Xsr (%)	STD	Xsr (%)	STD	Xsr (%)	STD	Xsr (%)	STD	Xsr (%)	STD
				**2**				**4**		**21**		**10**		**11**		**20**	
*S. aria*	*Aria*	Grkarica, Mt. Igman	2x	n.d.				n.d.		n.d.		n.d.		n.d.		n.d.	
		Bijela gora, Mt. Orjen	2x	n.d.				n.d.		n.d.		n.d.		n.d.		n.d.	
		Gradac, Posušje	2x	n.d.				n.d.		n.d.		n.d.		n.d.		n.d.	
		Grkarica, Mt. Igman	3x	n.d.				n.d.		n.d.		n.d.		n.d.		n.d.	
		Umoljani, Mt. Bjelašnica	3x	n.d.				n.d.		n.d.		n.d.		n.d.		n.d.	
		Bijela gora, Mt. Orjen	3x	n.d.				n.d.		n.d.		n.d.		n.d.		n.d.	
		Grkarica, Mt. Igman	4x	n.d.				n.d.		n.d.		n.d.		n.d.		n.d.	
		Umoljani, Mt. Bjelašnica	4x	n.d.				n.d.		n.d.		n.d.		n.d.		n.d.	
		Bijela gora, Mt. Orjen	4x	n.d.				n.d.		n.d.		n.d.		n.d.		n.d.	
*S. aucuparia*	*Sorbus*	Veliko polje, Mt. Igman	2x	n.d.				n.d.		n.d.		n.d.		n.d.		n.d.	
*S. austriaca*	*Soraria* (*Sorbus* × *Aria*)	Grkarica, Mt. Igman	4x	n.d.				n.d.		n.d.		n.d.		n.d.		n.d.	
		Umoljani, Mt. Bjelašnica	4x	n.d.				n.d.		n.d.		n.d.		n.d.		n.d.	
*S. bosniaca*		Mt. Krug planina	4x	n.d.				n.d.		n.d.		n.d.		n.d.		n.d.	
*S. torminalis* × *aria*	*Tormaria* (*Torminalis* × *Aria*)	Pratača, Lokve	2x	0.226 *		**0.015**		trace *	/	2.414 *	**0.013**	0.107 *	**0.013**	0.464 *	**0.005**	0.161 *	**0.01**
		Koznik	3x	0.059 *		**0.014**		n.d.		0.617 *	**0.092**	0.148 *	**0.012**	0.348 *	**0.006**	0.007 *	**0.001**
		Crne lokve, Posušje	4x	0.167 *		**0.015**		trace *	/	n.d.		0.206 *	**0.014**	0.347 *	**0.005**	1.224 *	**0.02**
*S. torminalis*	*Torminaria*	Tihovići	2x	n.d.				trace *	/	8.913	0.124	0.186 *	**0.013**	1.159 *	**0.020**	0.506 *	**0.007**
**Taxon**	**Subgenus**	**Location**	**DNA Ploidy Level**	**Flavonols**													
				Xsr (%)	STD	Xsr (%)	STD	Xsr (%)	STD	Xsr (%)	STD	Xsr (%)	STD	Xsr (%)	STD	Xsr (%)	STD
				**1**		**3**		**5**		**6**		**7**		**8**		**9**	
*S. aria*	*Aria*	Grkarica, Mt. Igman	2x	n.d.		n.d.		n.d.		n.d.		4.738	0.022	n.d.		1.779	0.011
		Mt. Bijela gora	2x	n.d.		n.d.		n.d.		n.d.		0.577	0.012	n.d.		2.847	0.005
		Gradac, Posušje	2x	n.d.		n.d.		n.d.		trace *	**/**	1.127	0.012	0.568	0.006	3.738	0.005
		Grkarica, Mt. Igman	3x	n.d.		n.d.		n.d.		n.d.		7.504	0.019	n.d.		1.791	0.007
		Umoljani, Mt. Bjelašnica	3x	n.d.		n.d.		0.878 *	**0.003**	trace *	**/**	3.555	0.008	0.249	0.007	1.586	0.003
		Mt. Bijela gora	3x	0.083 *	**0.005**	n.d.		n.d.		n.d.		3.715	0.027	0.305	0.008	1.266	0.014
		Grkarica, Mt. Igman	4x	n.d.		n.d.		n.d.		n.d.		4.115	0.044	0.138	0.005	1.898	0.016
		Umoljani, Mt. Bjelašnica	4x	n.d.		n.d.		n.d.		n.d.		4.115	0.044	0.138	0.005	1.898	0.016
		Mt. Bijela gora	4x	n.d.		n.d.		n.d.		n.d.		5.330	0.010	n.d.		2.004	0.007
*S. aucuparia*	*Sorbus*	Veliko polje, Mt. Igman	2x	n.d.		0.346	0.004	n.d.		n.d.		0.196	0.013	0.460	0.014	0.641	0.004
*S. austriaca*	*Soraria* (*Sorbus* × *Aria*)	Grkarica, Mt. Igman	4x	0.377 *	**0.009**	n.d.		n.d.		trace *	**/**	3.175	0.023	0.469	0.015	1.043	0.008
		Umoljani, Mt. Bjelašnica	4x	n.d.		trace		trace *	**/**	n.d.		1.960	0.029	0.223	0.011	1.345	0.017
*S. bosniaca*		Mt. Krug planina	4x	n.d.		n.d.		n.d.		0.179 *	**0.036**	3.695 *	**0.020**	2.658 *	**0.012**	3.102 *	**0.018**
*S. torminalis* × *aria*	*Tormaria* (*Torminalis* × *Aria*)	Pratača,Mt. Igman	2x	n.d.		n.d.		n.d.		0.223	0.010	0.267	0.021	1.896	0.014	1.883	0.011
		Koznik	3x	n.d.		n.d.		trace	/	0.098	0.003	1.594	0.020	1.592	0.014	0.939	0.009
		Crne lokve, Posušje	4x	n.d.		n.d.		n.d.		0.265	0.008	2.520	0.009	1.613	0.009	2.063	0.003
*S. torminalis*	*Torminaria*	Tihovići	2x	n.d.		n.d.		n.d.		n.d.		0.236	0.010	1.284	0.009	0.642	0.01
				**12**		**13**		**14**		**15**		**16**		**17**		**22**	
*S. aria*	*Aria*	Grkarica, Mt. Igman	2x	0.886 *	**0.005**	n.d.		2.582	0.015	n.d.		0.698	0.005	n.d.		0.366 *	**0.003**
		Mt. Bijela gora	2x	n.d.		0.167 *	**0.01**	n.d.		n.d.		n.d.		2.736	0.010	0.108 *	**0.003**
		Gradac, Posušje	2x	n.d.		0.131 *	**0.007**	0.650	0.003	n.d.		0.308	0.016	n.d.		n.d.	
		Grkarica, Mt. Igman	3x	0.459 *	**0.002**	n.d.		2.380	0.010	n.d.		0.263	0.003	n.d.		0.244 *	**0.005**
		Umoljani, Mt. Bjelašnica	3x	n.d.		0.435 *	**0.010**	0.783	0.004	n.d.		n.d.		1.853	0.003	trace *	**/**
		Mt. Bijela gora	3x	0.330 *	**0.048**	0.500 *	**0.012**	1.047	0.132	n.d.		n.d.		1.651	0.000	n.d.	
		Grkarica, Mt. Igman	4x	0.958 *	**0.012**	n.d.		1.323	0.031	n.d.		1.056	0.015	n.d.		0.231 *	**0.009**
		Umoljani, Mt. Bjelašnica	4x	0.209 *	**0.007**	n.d.		0.774	0.013	n.d.		0.136	0.006	n.d.		trace *	**/**
		Mt. Bijela gora	4x	1.033 *	**0.015**	n.d.		0.668	0.009	n.d.		0.624	0.008	n.d.		trace *	**/**
*S. aucuparia*	*Sorbus*	Veliko polje, Mt. Igman	2x	0.288	0.022	n.d.		0.331	0.005	n.d.		n.d.		n.d.		trace	/
*S. austriaca*	*Soraria* (*Sorbus* × *Aria*)	Grkarica, Mt. Igman	4x	n.d.		0.281 *	**0.006**	2.103	0.021	0.384 *	**0.003**	n.d.		n.d.		0.153 *	**0.009**
		Umoljani, Mt. Bjelašnica	4x	n.d.		0.128 *	**0.003**	1.609	0.012	0.222 *	**0.015**	n.d.		n.d.		0.082 *	**0.001**
*S. bosniaca*		Mt. Krug planina	4x	n.d.		0.590 *	**0.01**	n.d.		n.d.		n.d.		n.d.		0.152 *	**0.000**
*S. torminalis* × *aria*	*Tormaria* (*Torminalis* × *Aria*)	Pratača, Mt. Igman	2x	n.d.		n.d.		0.367	0.006	n.d.		n.d.		n.d.		0.198	0.001
		Koznik	3x	n.d.		0.353	0.013	0.780	0.010	n.d.		n.d.		n.d.		n.d.	
		Crne lokve, Posušje	4x	0.346	0.039	n.d.		0.431	0.004	n.d.		n.d.		n.d.		n.d.	
*S. torminalis*	*Torminaria*	Tihovići	2x	n.d.		n.d.		n.d.		n.d.		0.339	0.008	n.d.		n.d.	
**Taxon**	**Subgenus**	**Location**	**DNA Ploidy Level**	**Methylflavonols**													
				Xsr (%)			STD		Xsr (%)		STD			Xsr (%)		STD	
				**18**					**19**					**23**			
*S. aria*	*Aria*	Grkarica, Mt. Igman	2x	n.d.					1.016 *		**0.006**			0.444 *		**0.005**	
		Mt. Bijela gora	2x	n.d.					n.d.					0.134 *		**0.002**	
		Gradac, Posušje	2x	n.d.					0.476 *		**0.009**			n.d.			
		Grkarica, Mt. Igman	3x	n.d.					0.724 *		**0.003**			0.205 *		**0.007**	
		Umoljani, Mt. Bjelašnica	3x	n.d.					n.d.					n.d.			
		Mt. Bijela gora	3x	n.d.					n.d.					n.d.			
		Grkarica, Mt. Igman	4x	n.d.					0.496 *		**0.021**			0.087 *		**0.008**	
		Umoljani, Mt. Bjelašnica	4x	n.d.					0.172 *		**0.002**			n.d.			
		Mt. Bijela gora	4x	n.d.					0.173 *		**0.003**			n.d.			
*S. aucuparia*	*Sorbus*	Veliko polje, Mt. Igman	2x	n.d.					n.d.					n.d.			
*S. austriaca*	*Soraria* (*Sorbus* × *Aria*)	Grkarica, Mt. Igman	4x	n.d.					n.d.					n.d.			
		Umoljani, Mt. Bjelašnica	4x	n.d.					n.d.					trace *		**/**	
*S. bosniaca*		Mt. Krug planina	4x	0.881 *			**0.007**		0.186 *		**0.003**			n.d.			
*S. torminalis* × *aria*	*Tormaria* (*Torminalis* × *Aria*)	Pratača, Mt. Igman	2x	0.833			0.014		0.738		0.014			n.d.			
		Koznik	3x	0.710			0.008		0.525		0.021			n.d.			
		Crne lokve, Posušje	4x	0.427			0.008		0.386		0.007			n.d.			
*S. torminalis*	*Torminaria*	Tihovići	2x	n.d.					1.669		0.015			n.d.			

Xsr—mean value of triplicates; STD—standard deviation; DE—dry extract. The names of the secondary metabolites correspond to their numerical values, which are given by Table 1: UV and MS spectral data of the detected flavonoids in EtOAc fractions of *Sorbus* accessions. Newly identified compounds from the leaves of *Sorbus* accessions are highlighted using bold font and marked with asterisk (*).

**Table 3:** Geographic origin, genome size and ploidy level of studied *Sorbus* accessions.

Taxon	Subgenus	Genome Size (2C pg)	DNA Ploidy Level	Location	Voucher Number	North	East	Altitude (m)
*S. aria*	*Aria*	1.47	2x	Grkarica, Mt. Igman	SARA (54276)	43.739167	18.291389	1350
		1.41	2x	Mt. Bijela gora	SARA (54280)	42.677778	18.475833	730
		1.41	2x	Gradac, Posušje	SARA (54278)	42.425	17.3925	720
		2.12	3x	Grkarica, Mt. Igman	SARA (51415)	43.739167	18.291389	1350
		2.11	3x	Umoljani, Mt. Bjelašnica	SARA (51412)	43.664167	18.226111	1300
		2.11	3x	Mt. Bijela gora	SARA (54277)	42.677778	18.475833	730
		2.82	4x	Grkarica, Mt. Igman	SARA (51416)	43.739167	18.291389	1350
		2.80	4x	Umoljani, Mt. Bjelašnica	SARA (51413)	43.664167	18.226111	1300
		2.78	4x	Mt. Bijela gora	SARA (54279)	42.677778	18.475833	730
*S. aucuparia*	*Sorbus*	1.43	2x	Veliko polje, Mt. Igman	SARA (51417)	43.745278	18.275	1210
*S. austriaca*	*Soraria* (*Sorbus* × *Aria*)	2.81	4x	Grkarica, Mt. Igman	SARA (51418)	43.739167	18.291389	1350
		2.77	4x	Umoljani, Mt. Bjelašnica	SARA (51414)	43.664167	18.226111	1300
*S. bosniaca*		2.80	4x	Mt. Krug planina	WU (080424)	43.842222	17.199722	1300
*S. torminalis* × *aria*	*Tormaria* (*Torminalis* × *Aria*)	1.40	2x	Pratača, Mt. Igman	SARA (54281)	43.763611	18.19138	915
		2.18	3x	Koznik	SARA (54282)	43.7125	17.9675	900
		2.74	4x	Crne lokve, Posušje	SARA (54283)	43.4425	17.464444	700
*S. torminalis*	*Torminaria*	1.44	2x	Tihovići	SARA (54284)	43.923889	18.377778	910

## Data Availability

All data generated during this study are included in this article.

## Supplementary Materials

The following supporting information can be downloaded at https://www.mdpi.com/article/10.3390/plants14010119/s1, Table S1. Calibration curves regression equations, correlation coefficients (r2), linear ranges, LODs and LOQs of reference compounds, Table S2. Principal components (PC) revealed by Principal Component Analysis (PCA) for the studied *Sorbus* accessions.
