# Supplementary material for: Phytochemical Characterisation of Sorbus Species: Unveiling Flavonoid Profiles Related to Ploidy and Hybrid Origin
Source: Plants (Basel). 2025 Jan 3;14(1):119. doi: 10.3390/plants14010119 (PMC11722658; doi:10.3390/plants14010119)
Supplement: Supplementary file 1 [file plants-14-00119-s001.zip › Table S2.pdf]

Table S2. Principal components (PC) revealed by Principal Component Analysis (PCA) for the studied *Sorbus* accessions.

**Compound**	**PC 1**	**PC 2**	**PC 3**
Hydroxyquercetin deoxyhexosyl hexoside	-0.08	0.16	-0.28
Apigenin 6,8-di-C-glucoside	0.29	0.03	0.01
Quercetin dihexoside	-0.06	0.00	-0.21
Apigenin-6-*C*-glucoside-8-*C*-arabinoside	0.32	-0.13	-0.02
Quercetin trideoxyhexoside	-0.03	0.21	-0.10
Quercetin deoxyhexosyl hexoside	0.25	0.21	0.06
Quercetin 3-*O*-rutinoside	-0.03	0.09	0.44
Quercetin 3-*O*-galactoside	0.30	0.14	-0.09
Quercetin-3-*O*-glucoside	0.35	-0.07	-0.08
Luteolin 7-*O*-glucoside	0.34	-0.11	-0.11
Luteolin 7-*O*-glucuronide	-0.13	-0.30	0.08
Kaempferol deoxyhexosylhexoside	-0.03	0.42	-0.04
Quercetin hexosylpentoside	-0.19	-0.10	-0.21
Quercetin acetylhexoside	0.15	0.20	0.41
Quercetin pentoside	-0.09	0.14	-0.28
Kaempferol 3-*O*-glucoside	-0.09	-0.39	0.09
Quercetin 3-*O*-rhamnoide	-0.07	0.27	0.11
Methylquercetin hexoside isomer 1	0.18	0.22	0.35
Methylquercetin hexoside isomer 2	0.17	-0.36	0.11
Luteolin hexoside	0.31	-0.10	-0.03
Apigenin 7-*O*-glucuronide	0.27	-0.13	-0.11
Kaempferol acetylhexoside	-0.16	-0.03	0.24
Methylquercetin acethylhexoside	-0.16	-0.18	0.29
