# Supplementary material for: Phytochemical Characterisation of Sorbus Species: Unveiling Flavonoid Profiles Related to Ploidy and Hybrid Origin
Source: Plants (Basel). 2025 Jan 3;14(1):119. doi: 10.3390/plants14010119 (PMC11722658; doi:10.3390/plants14010119)
Supplement: Supplementary file 1 [file plants-14-00119-s001.zip › Table_S1.pdf]

Table S1. Calibration curves regression equations, correlation coefficients (r^2), linear ranges, LODs and LOQs of reference compounds.

**Regression equation**	**r^2**	**linear range (μg/mL)**	**LOD (μg/mL)**	**LOQ (μg/mL)**	**Compound**
y = 4066.8457x + 47.2773	0.9999	0.16-250	0.022	0.087	Apigenin 6,8-di-*C*-glucoside
y = 4128.7213x + 15.5276	0.9998	0.62-500	0.029	0.110	Apigenin-6-*C*-glucoside-8-*C*-arabinoside
y = 2755.8034x + 22.2383	0.9999	0.16-250	0.011	0.041	Quercetin 3-*O*-rutinoside
y = 3450.5525x + 19.4631	0.9999	0.62-500	0.027	0.110	Quercetin 3-*O*-galactoside
y = 7039.2891x + 22.7091	0.9999	0.62-500	0.035	0.139	Quercetin-3-*O*-glucoside
y = 7110.2527x - 79.8848	0.9996	0.16-250	0.021	0.082	Luteolin 7-*O*-glucoside
y = 5020.3480x - 2.7332	0.9999	0.62-500	0.031	0.124	Luteolin 7-*O*-glucuronide
y = 4007.6421x + 5.7835	0.9999	0.16-250	0.031	0.121	Kaempferol 3-*O*-glucoside
y = 2941.0244x + 7.5674	0.9999	0.62-500	0.033	0.133	Quercetin 3-*O*-rhamnoide
y = 4514.5221x - 9.4910	0.9995	0.62-500	0.027	0.110	Apigenin 7-*O*-glucuronide
